# Impact of COVID-19 pandemic lockdown on exclusive breastfeeding in non-infected mothers

**DOI:** 10.1186/s13006-021-00382-4

**Published:** 2021-04-17

**Authors:** Giuseppe Latorre, Domenico Martinelli, Pietro Guida, Ester Masi, Roberta De Benedictis, Luca Maggio

**Affiliations:** 1 Neonatology and Neonatal Intensive Care Unit, Ecclesiastical General Hospital F. Miulli, Acquaviva delle Fonti, Italy; 2 Department of Woman and Child Health and Public Health, Child Health Area, Fondazione Policlinico Universitario A. Gemelli, IRCCS, Università Cattolica del Sacro Cuore, Rome, Italy

**Keywords:** COVID-19, Breastfeeding, Coronavirus, Mother-infant dyad, Home confinement, Neonatal, Lockdown, Pandemic

## Abstract

**Background:**

The COVID-19 pandemic has posed several challenges to the provision of newborn nutrition and care interventions including maternal support, breastfeeding and family participatory care. Italy was the first country to be exposed to SARS-CoV-2 in Europe. One of the measures adopted by the Italian government during COVID-19 pandemic was the total lockdown of the cities with complete confinement at home. We aimed to examine the impact of the lockdown caused by COVID-19 pandemic on exclusive breastfeeding in non-infected mothers.

**Methods:**

We prospectively enrolled 204 mother-baby dyads during lockdown (9 March to 8 May 2020) that we compared to previously studied 306 mother-baby dyads admitted during the year 2018. To reduce the possible effect of confounding factors on exclusive breastfeeding, a 1:1 matching was performed by using an automatized procedure of stratification that paired 173 mother-baby dyads. Feeding modality was collected at discharge, 30 and 90 days of newborn’s life. Exclusive breastfeeding was considered when the infant received only breast milk and no other liquids or solids were given with the exception of vitamins, minerals or medicines.

**Results:**

At discharge 69.4% of infants were exclusively breastfed during lockdown versus 97.7% of control group, 54.3% at 30 days vs 76.3 and 31.8% vs 70.5% at 90 days (*p* < 0.001). The proportion of breastfeeding remaining exclusive from discharge to 30-day was similar between groups (about 80%), but it was lower in lockdown group than in control cohort (58.5% vs 92.4%, *p* < 0.001) from 30- to 90-days.

**Conclusions:**

Lockdown and home confinement led to a decrease of exclusively breastfeeding in the studied population. Considering the timing to shift from exclusive to non-exclusive breastfeeding, differences between study groups were concentrated during hospital stay and from 30- to 90 days of a newborn’s life, confirming that the hospital stay period is crucial in continuing exclusive breastfeeding at least for the first 30 days, but no longer relevant at 90 days of life.

**Supplementary Information:**

The online version contains supplementary material available at 10.1186/s13006-021-00382-4.

## Background

The pandemic caused by novel Severe Acute Respiratory Syndrome Coronavirus 2 (SARS-CoV-2) isolated in Wuhan, China at the end of 2019 led to a disruption of the daily activities of the population and struck Italy with major public health and economic consequences. Italy was one of the first countries to be exposed to the SARS-CoV-2 and was the first country in Europe to be affected by the pandemic. To contain the spread of the virus and to avoid the health system to collapse, the Italian government released (9 March 2020) an executive order to implement a state of alarm. One of the measures adopted by the Italian government was the total lockdown of the cities with complete confinement at home, with the closure of factories, the reduction of both private and public transportation, vacation for schools, and working from home [1, 2]. Many hospitals changed their usual practices. Despite birth being perceived as one of the most important life events, hospitals did not allow partners to be present during labour and no visitors were allowed in the postpartum ward. In many hospitals only one visitor per day could visit an infant in the neonatal units, which might lead to long term neonatal bonding problems as well as parental psychosocial complications and depression. After discharge the new family remained isolated from family and friends owing to social distancing rules [3].

Early in the COVID-19 pandemic, limited data existed regarding the risk of adverse outcomes for pregnant women infected with SARS-CoV-2, and the risk of vertical or horizontal transmission to their newborn was unknown [4–6]. Best practices around breastfeeding during maternal COVID-19 infection has been compromised by the lack of rigorous evidence as to whether SARS-CoV-2 can be vertically transmitted in milk or during breastfeeding [7–10]. The COVID-19 pandemic has posed several challenges to the provision of newborn nutrition and care interventions, including maternal support, breastfeeding, kangaroo mother care, and family participatory care. Both the World Health Organization (WHO) and United Nations Children’s Fund (UNICEF) recommend early initiation of breastfeeding and exclusive breastfeeding during the first 6 months of life. Breastfeeding is associated with improved infant survival and significant health benefits both for infants and mothers. Promotion and support of breastfeeding initiation, duration, and exclusivity is a public health issue [11, 12]. A position statement of the Italian Society of Neonatology invited clinicians to promote breastfeeding in all women even those affected by SARS-CoV-2 [13, 14]. The first lockdown in Italy lasted 2 months. Epidemiological studies showed that COVID-19 pandemic has modified dietary trends of adolescents from many countries and has increased psychiatric disorders such as depressive and anxiety disorders and habits that could have some later impact on health [15–17]. There are no studies on the impact of lockdown measure on breastfeeding. We aimed to examine the impact of the lockdown caused by COVID-19 pandemic on exclusive breastfeeding in non-infected mothers.

## Methods

We conducted a single-center study in the nursery of Ecclesiastical General Hospital F. Miulli in Acquaviva delle Fonti, Italy. Consecutive mother-baby dyads admitted to the unit during lockdown from 9 March to 8 May 2020 (lockdown group) were prospectively evaluated. As the control group, we considered a retrospective population of mother-baby dyads admitted during the year 2018 [18]. The healthcare personal involved in mother-baby dyads care was the same in the two study periods.

Healthy newborns with a gestational age ≥ 37 weeks, “rooming in” (baby in the same room of the mother all day) from birth to discharge and never hospitalized in Neonatal Intensive or Sub-Intensive Care Unit were included. Exclusion criteria were all maternal and/or neonatal conditions that could interfere with breastfeeding (maternal HIV or active tuberculosis infection, herpes simplex lesions on both breasts, use of therapeutic radioactive isotopes, or exposure to radioactive materials, galactosemia of the infant) or women not speaking Italian to ensure a full understanding of the questionnaire. Informed consent was obtained from both parents.

During lockdown period, 257 infants were born: 31 with gestational age < 37 weeks and 16 not assisted in rooming were excluded. A total of 210 dyads met the eligibility criteria. Among those, six were excluded (three declined to participate and three did not speak Italian). In the lockdown group a total of 204 mother-baby dyads were enrolled. Data were compared to previously studied 306 mother-baby dyads admitted during the year 2018. To reduce the possible effect of confounding factors on exclusive breastfeeding, a 1:1 matching was performed by using an automatized procedure of stratification that paired 173 mother-baby dyads according to infant’s gender, maternal schooling, prenatal class attendance and caesarean section by selecting mothers with a more similar maternal age at delivery. Additional figures show the flowchart of mother-baby selection.

At discharge, a structured interview was performed, and a questionnaire was administered to the mother. Data were collected by a healthcare professional at discharge or extracted from the infants’ computerized medical charts (Neocare, I&T Informatica e Tecnologia Srl, Italy). The variables investigated included sociodemographic features (maternal age, education), previous experiences (participation to a prenatal class and previous pregnancy), type of delivery, use of pacifier, and nipple fissures. Variables subjected to changes during the timeframe of the study were collected by phone interview at 30 and 90 days of the newborn’s life. The mode of breastfeeding was defined according to World Health Organization (WHO) definition and changes over time were reported [19]. Exclusive breastfeeding meant that the infant received only breast milk; no other liquids or solids were given with the exception of vitamins, minerals or medicines.

### Statistical analysis

Data are reported as mean ± standard deviation or percentage for categorical variables. We used the Student’s t-test to compare continuous variables and chi-squared test to evaluate associations between categorical data. Conditional logistic regression, appropriate for matched data, were used to analyze paired sub-samples. A *p* - value of 0.05 or less was considered statistically significant. All analyses were conducted using Stata software, version 16 (Stata-Corp LP, College Station, Texas).

## Results

Table 1 shows characteristics of mothers and infants in the overall population, in the lockdown group, in the control group and in the sub-samples of mothers and infants matched by stratification. There were differences between the two cohorts in terms of maternal education (more primary than secondary education during lockdown period), prenatal class attendance (less frequently observed in the lockdown group). No difference was detected by study groups for maternal age at delivery, parity, gestational age, gender and weight of infants. Matched groups had similar characteristics.

**Table 1:** Characteristics of participants

	Overall				Paired by stratification		
	All ***n*** = 510	Control ***n*** = 306	Lockdown ***n*** = 204	***p***	Control ***n*** = 173	Lockdown ***n*** = 173	***p***
Maternal age at delivery (years)	33 ± 5	33 ± 5	33 ± 5	0.726	33 ± 5	33 ± 5	0.426
Maternal education				0.008			–
Primary school	11.4%	7.8%	16.7%		12.1%	12.1%	
Secondary education	53.1%	56.2%	48.5%		48.6%	48.6%	
University degree	35.5%	35.9%	34.8%		39.3%	39.3%	
Prenatal class	42.2%	50.7%	29.4%	< 0.001	32.9%	32.9%	–
Parity	1.5 ± 0.6	1.5 ± 0.5	1.5 ± 0.7	0.351	1.6 ± 0.5	1.5 ± 0.7	0.367
Gestational age (weeks)	39 ± 1	39 ± 1	39 ± 1	0.375	39 ± 1	39 ± 1	0.712
Caesarean section	32.4%	27.8%	39.2%	0.007	33.5%	33.5%	–
Male infant	48.8%	46.7%	52.0%	0.247	49.1%	49.1%	–
Infant weight at birth (g)	3321 ± 428	3330 ± 424	3307 ± 435	0.558	3338 ± 417	3308 ± 423	0.477
Infant weight at discharge (g)	3122 ± 413	3128 ± 404	3113 ± 428	0.703	3140 ± 407	3112 ± 416	0.526
Nipple fissure	22.2%	31.4%	8.3%	< 0.001	28.3%	9.8%	< 0.001
Pacifier	43.9%	42.8%	45.6%	0.536	43.4%	46.8%	0.527

Mean ± Standard Deviation or percentage

**Fig. 1:**
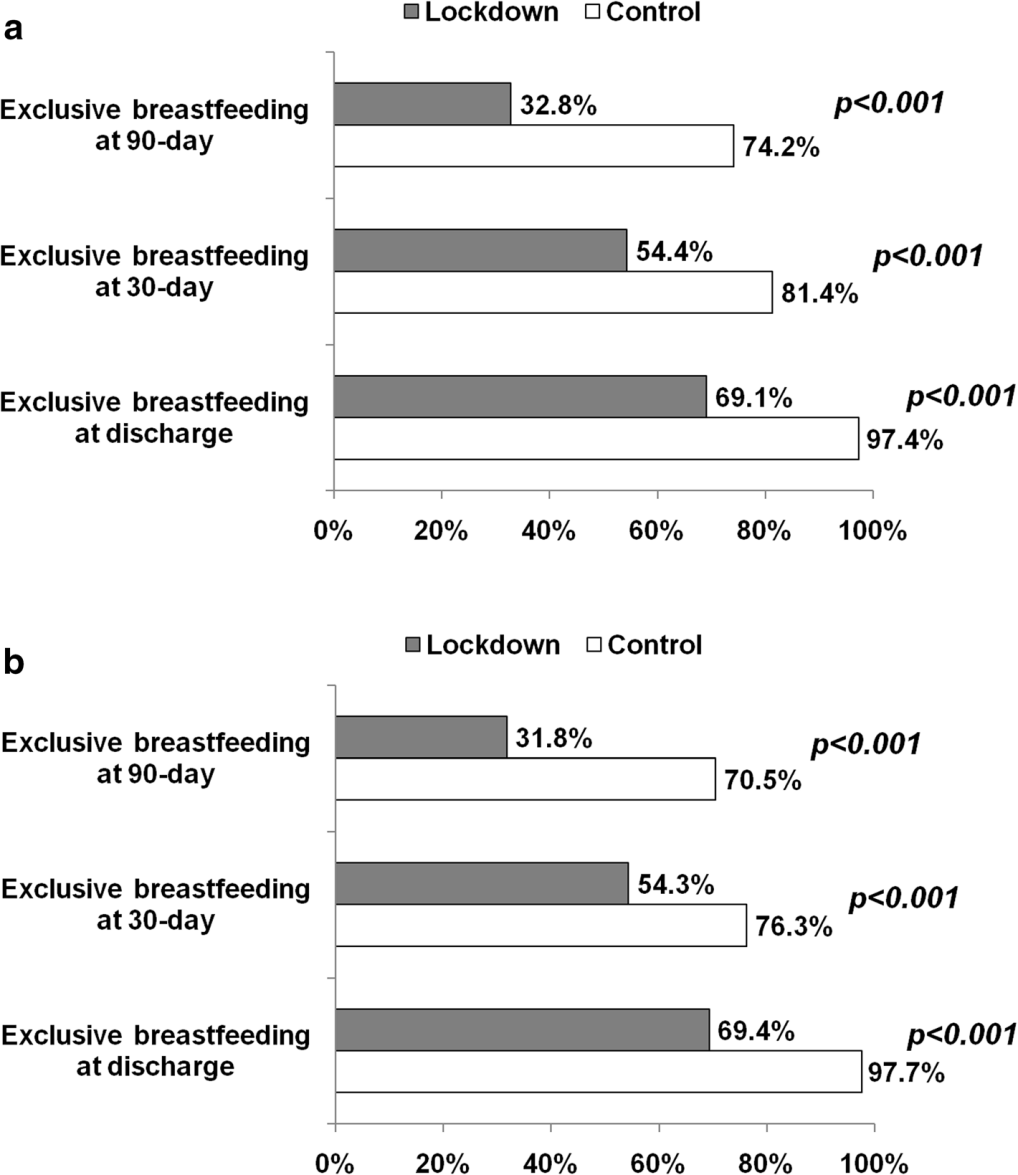
**a**: Proportion of exclusive breastfeeding at discharge, at 30 and 90 days of life in lockdown and control group. **b**: Proportion of exclusive breastfeeding at discharge, 30 and 90 days of life in lockdown and control group after stratification to pair data (1:1 control and lockdown period)

Figure 1 shows the proportion of exclusive breastfeeding at discharge, 30- and 90-day in the lockdown and in the control group (panel A) and in the paired populations (panel B). At each time-point, exclusive breastfeeding was less frequent in lockdown than control period with a temporal reduction trend in both study groups. The use of infant formula feeding was higher during the lockdown than control period. At 90 days, exclusive breastfeeding was more frequent in control than lockdown group (74.2% vs 32.8%, Fig. 1a).

**Fig. 2:**
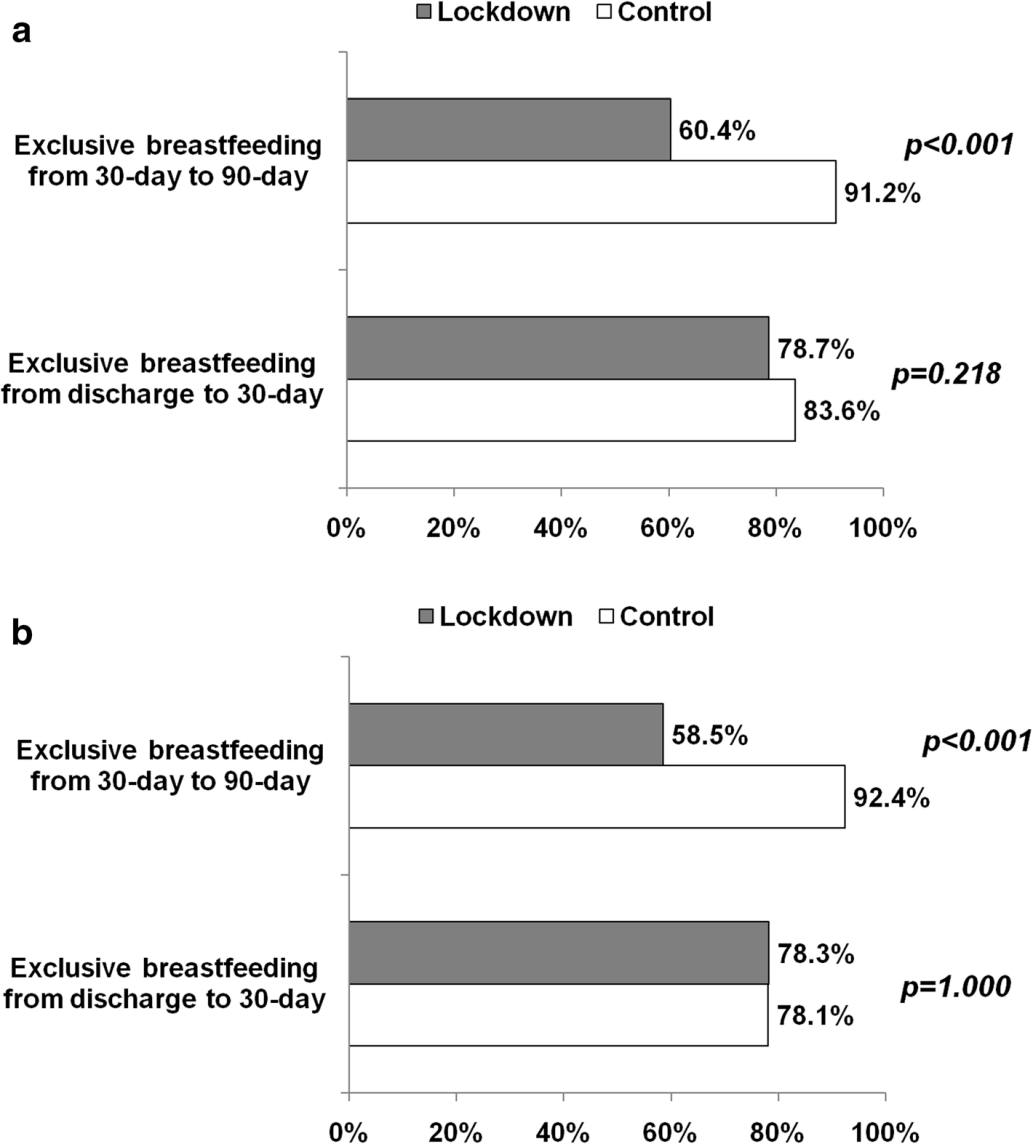
**a**: Proportion of exclusive breastfeeding during the intervals 0–30 days and 30–90 days in lockdown and control group. **b**: Proportion of exclusive breastfeeding during the intervals 0–30-days and 30–90 days in lockdown and control group after stratification to pair data (1:1 control and lockdown period). The proportion was computed considering infants in exclusive breastfeeding at the beginning of period (at discharge and at 30-day)

We analysed separately two periods: from discharge to 30 days and thereafter up to 90 days. Among infants discharged in exclusive breastfeeding, approximately 80% remained exclusive breastfed at 30 days without differences between groups. However, from 30 to 90 days, the proportion of infants remaining in exclusive breastfeeding was lower in the lockdown than control group in both overall and paired populations (Fig. 2a and b).

The use of pacifier was similar in the two periods; the incidence of nipple fissure was higher in mothers from control group than those in lockdown period.

## Discussion

Exclusively breastfeeding improves the survival, health and development of all children. The advantages of exclusive breastfeeding have been well studied and they are universally recognized [20–22]. Many factors contribute to the initiation and continuation of breastfeeding. Women giving birth by a planned caesarean section and women with psychological stress conditions during delivery are at greater risk for shorter exclusively breastfeeding duration [23, 24]. In our study the rate of exclusively breastfeeding at discharge was statistically low during lockdown than in control group. The caesarean section rate for women with confirmed COVID-19 infection has been reported as ranging from 42 to 92% in other studies [25]. There are no data about caesarean section during lockdown for COVID-19 pandemic in non-infected pregnant women. The higher caesarean procedures found in our population, could be not only in maternal interest but also concern for maternal access to hospital care due to limitation of transportation. Even if the mode of delivery modulates breastfeeding success, it must be considered that, in our study, caesarean section does not seem to negatively impact breastfeeding more than lockdown itself. We performed a matched analysis to isolate the possible effect of several confounders on cessation of exclusive breastfeeding. The lower proportion of exclusive breastfeeding during the first 90 days of a newborn’s life in the lockdown group was detected in matched data, having a good balanced of mother-baby dyads characteristics and delivery method.

Antenatal and postnatal support, including mothers’ counselling and education, have been shown to positively affect breastfeeding success [21, 26]. However, our results show that the low participation of mothers to prenatal class in the lockdown group does not appear to be a confounding factor of the lower exclusive breastfeeding, as shown by the analysis of paired dyads.

The first few hours and days of a newborn’s life are a critical window for establishing lactation and for providing mothers with the support they need to breastfeed successfully [19]. Health professional support during hospital stay and after hospital discharge was associated with a decreased risk of exclusively breastfeeding cessation [12]. In our study considering the timing to shift from exclusive to non-exclusive breastfeeding, differences between study groups were concentrated during hospital stay and from 30- to 90-day and not from discharge to 30 days of life, confirming that the hospital stay period is crucial in continuing exclusive breastfeeding at least for the first 30 days of a newborn’s life, but no longer relevant at 90 days of life.

Disasters are devastating for anyone affected, but pregnant and breastfeeding women often have specific concerns about the effects of certain exposures (such as infections, chemicals, medications, and stress) on their fetus or breastfed child [27–29]. The COVID-19 pandemic has posed several challenges to the provision of newborn nutrition and care interventions, including maternal support, breastfeeding, and family participatory care. Mothers and healthcare workers are confused and scared of COVID-19 and breastfeeding, along with a sense of heightened stigma about the disease which is impacting access to breastmilk [10, 30]. Restrictions in lockdown made it difficult for mothers and families to get access to feeding support. The availability of adequate support at the community level is associated with exclusive breastfeeding at 3 months post-delivery [12], unfortunately during the lockdown, the new family was isolated from family and friends owing to social distancing rules.

It is the first time that Italian population faced such a government measure as lockdown and home confinement. During hospital stay all pregnant women and healthcare professional had to wear individual protection dispositive: masks, gloves and sometimes protective glasses and the feeling that any person encountered was “suspicious”. Therefore, it is vital that neonatal nurses, midwives and other healthcare professionals are adequately informed and educated about the potential impact of lockdown on neonatal practice [31].

According to our data, the imposition of lockdown during the COVID-19 pandemic seems have had a negative impact on breastfeeding. Promotion and support of breastfeeding initiation, duration, and exclusivity is a public health issue [11, 12]. In the studied population we found that lockdown and home confinement led to a decrease of exclusively breastfeeding. This should be considered to improve and promote strategies to help mother during pregnancy and after delivery in such situations. We did not address the study to explain the causes of the reduction of exclusive breastfeeding in infants born during lockdown in Italy.

The limit of our study is that it is a single center study and could be affected by local policy. Moreover, the mother-baby dyads were enrolled during the lockdown period (about 2 months), so that its influence on breastfeeding duration for the next 90 days after enrolment was different for each dyad, and different causes may have interfered with the reduced percentage of exclusive breastfeeding from 30-day to 90-days. Further research is needed, in order to replicate this study in other settings in which the government measure to stop COVID-19 pandemic was the lockdown, as was done in Italy. The most pressing issue currently is a need for more tailored guidance and specific considerations for early care and education programs during the COVID-19 pandemic that address pregnant women and newborn health [32]. A comprehensive newborn nutrition and care response comprising of optimal lactation support is needed to improve neonatal health outcomes. Globally, there are efforts to address such challenges. This study might be useful to improve the response to the unique needs of this special population of the mother-baby dyads and may suggest the importance of providing breastfeeding support to women during any public health emergency.

## Conclusions

In this study we observed that lockdown and home confinement led to a decrease of exclusively breastfeeding. This finding suggests the importance of providing support to breastfeeding women during any public health emergency to avoid breastfeeding cessation.

## Supplementary Information

**Additional file 1.** Flowchart of participant selection.

## Data Availability

The dataset could be obtained from the corresponding author upon reasonable request.
