# Supplementary figures and images for: Impact of COVID-19 pandemic lockdown on exclusive breastfeeding in non-infected mothers
Source: Int Breastfeed J. 2021 Apr 17;16:36. doi: 10.1186/s13006-021-00382-4 (PMC8052849; doi:10.1186/s13006-021-00382-4)

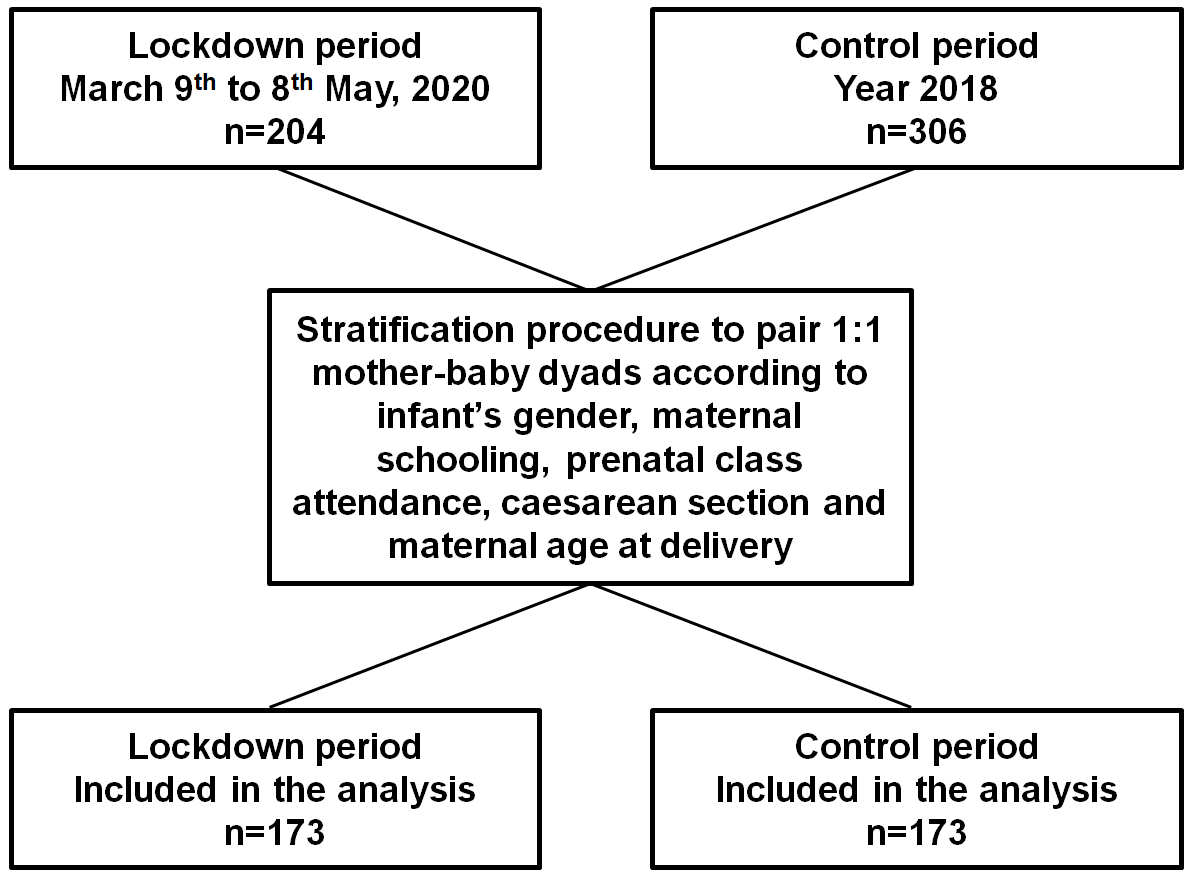

Supplement: Supplementary file 1 — Additional file 1. Flowchart of participant selection. [file 13006_2021_382_MOESM1_ESM.tif]
